# *Insilico* analyses of heparin binding proteins expression in human periodontal tissues

**DOI:** 10.1186/s13104-016-1857-1

**Published:** 2016-01-28

**Authors:** Bernadette Lackey, Quentin M. Nunes, Susan M. Higham, David G. Fernig, Sabeel P. Valappil

**Affiliations:** Department of Health Services Research and School of Dentistry, University of Liverpool, Research Wing, Daulby Street, Liverpool, L69 3GN UK; NIHR Liverpool Pancreas Biomedical Research Unit, Royal Liverpool University Hospital, Daulby Street, Liverpool, L69 3GA UK; Department of Structural and Chemical Biology, Institute of Integrative Biology, University of Liverpool, Crown Street, Liverpool, L69 7ZB UK

**Keywords:** Periodontitis, Heparin, Heparan sulfate, Matrixmetalloproteinase

## Abstract

**Background:**

Periodontitis is described as a group of inflammatory diseases of the gingiva and supporting structures of the periodontium. The accumulation of plaque bacteria, which include putative periodontal pathogens, is known to initiate the disease but the host immune response is the major contributing factor for destruction of periodontal tissues. Proteins that bind to heparin heparin-binding protein (HBPs) play important roles in health and disease and interact with each other via networks known as ‘heparin interactomes’. This study aimed at evaluating published datasets of HBPs and its role in periodontitis.

**Methods:**

To elucidate the role of HBPs in periodontitis, bioinformatics analyses of published data was used. *In silico* analyses of published datasets were used to construct a putative HBPs interactome using an online database resource, ‘STRING’ (Search Tool for the Retrieval of Interacting Genes).

**Results:**

PubMed searches identified 249 genes that were up regulated and 146 genes that were down regulated in periodontal disease, compared with periodontal disease-free gingival samples. *In silico* analyses using published datasets revealed 25 up-regulated and 23 down-regulated HBPs in periodontitis. Of these HBPs; chemokines, such as CXCL12 was up regulated where as some of the matrixmetalloproteinases (MMPs; MMP-2 and MMP9) were up-regulated while MMP-14 was down regulated.

**Conclusions:**

The results indicate that HBP analyses will provide multiple targets for the biological mechanisms underlying periodontal disease (such as MMPs, cytokines and chemokines) that will have important clinical implications in the future drug design and management of periodontal disease.

**Electronic supplementary material:**

The online version of this article (doi:10.1186/s13104-016-1857-1) contains supplementary material, which is available to authorized users.

## Background

Periodontitis is a multifactorial disease instigated by the accumulation of certain pathogenic plaque bacteria that leads to the damage of the supporting tissues of teeth and can affect up to 45 % of UK dentate adults [1]. This is a disturbing development, as periodontitis may be a risk factor for severe systemic conditions such as arteriosclerosis, myocardial infarction and stroke; preterm, low birth weight babies and pose threats to those with chronic disease such as diabetes, respiratory diseases and osteoporosis [2]. Currently, periodontal therapy involves scaling or root planning, and in more severe cases antimicrobial agents such as doxycycline, metronidazole, minocycline or combined antimicrobial chemotherapy. While drug treatment can result in control of pain and swelling, it is difficult to stop the associated structural destruction. Thus, attention has been channelled to finding ways to inhibit the biological mechanisms that underlie the inflammation process.

To date, several proteins influencing periodontitis have been identified but how these proteins interact with each other in the progression of periodontitis is still not clear. Therefore identifying such interactions will be useful in determining the target towards therapeutic development. From this angle, heparin-binding proteins (HBPs) which are extracellular regulatory proteins that mediate cell communication in development, homeostasis and disease [3–5] appear to be very important in the understanding of the progression of periodontitis. It has been reported that HBPs such as azurocidin could be a potential candidate for a biomarker for the early detection of inflammatory periodontal destruction [6]. Many pathogens express proteins such as matrixmetalloproteinases (MMPs) that interact with heparin/heparan sulfate (HS), as part of their molecular adaptation to infection of mammals [7].

Fibroblasts secrete collagenase MMPs causing periodontium degradation, whilst fibronectin, inhibits expression of interleukin-1 and modulates this pathogenic mechanism [8]. MMPs are endopeptidases that require metal ions as cofactors for activity and are critical in collagenous cartilage matrix degradation [9]. MMPs are responsible for the destruction of collagen (MMP- 1 and 8), stromolysins (MMP-3, 10 and 11) for that of proteoglycans [10]. Tissue inhibitors of MMPs, TIMPs counteract the destructive effect of MMPs, and alterations of this balance causes pathological destruction of the periodontium [10]. These proteins are HBPs [4], which may explain in part the effects of heparin in periodontitis, where it alters MMP/TIMP complexes circulating in blood, and increases release of TIMP-2 [11]. Heparin/HS, HBPs and MMPs are, thus, important in periodontitis. But detailed studies have not been conducted to analyse such interations in periodontitis. Therefore the aim of this study was to integrate and rationalise available data on HBPs with a view to identify drug targets that play important roles in periodontitis.

## Methods

### Construction of the heparin-binding putative protein interactome in periodontitis and network analysis

HBPs associated with periodontitis were obtained using a combination of searches in PubMed using search terms such as ‘periodontal disease’, ‘periodontitis’, ‘periodontal disease microarray’, ‘periodontitis microarray’, ‘periodontitis and heparin’, periodontal disease and heparin’, ‘periodontitis and ‘heparan sulphate’ and ‘periodontal disease and ‘heparan sulphate’. A PubMed search identified 249 genes that were up regulated and 146 genes that were down regulated in periodontal disease, compared with periodontal disease-free gingival samples (supplementary information, [4]). Interactions between HBPs in periodontitis were obtained using the online database resource ‘Search Tool for the Retrieval of Interacting Genes’ (STRING), as described previously for analogous datasets of HBPs in normal pancreas and pancreatic disease [5]. STRING 9.1 is a database of known and predicted functional interactions and is a comprehensive resource that can be used with Cytoscape [12]. In STRING interactions are given a confidence score that estimates the likelihood of the interaction describing a functional linkage between the two proteins. Only interactions with the highest confidence score (0.900 and above) were used to build networks using Cytoscape 2.8.1, [13]. The connectivity networks were called ‘putative protein interactomes’, because the HBP lists were derived from mRNA expression data and the interactions between the HBPs were obtained from STRING. The network parameters were analysed using the *‘NetworkAnalyzer’* plugin [14] available in Cytoscape. The *‘Cluster ONE’* plugin in Cytoscape was used to identify densely connected, cohesive groups of HBPs within the putative HBP interactome, with a view to identifying potential drug targets in periodontitis [15]. In the putative protein interactome graphs, the HBPs or ‘nodes’ are connected by black lines that denote the interactions or ‘edges’.

### Functional analysis of HBPs

Tools for gene ontology (GO) term enrichment were used to undertake functional analyses of the HBPs in periodontitis, as described previously [5]. GO covers biological process (BP), cellular component (CC) and molecular function (MF) sub-ontologies. Enrichment of GO terms is a means to provide biological context to the datasets of HBPs. It was performed using the ‘Database for annotation, visualization and integrated discovery’ (DAVID) and GO FAT annotation [16]. GO FAT is a subset of the GO term set created by filtering out the broadest ontology terms to avoid overshadowing more specific ones.

## Results and discussion

### Construction of the heparin-binding putative protein interactome in periodontitis and network analysis

HBPs play a major role in many fundamental biological processes in health and disease [3, 4]. The potential of HBPs as therapeutic targets in periodontal disease is evident from the recent use of an HS-mimetic to facilitate regeneration of the periodontium in the presence of pathogenic periodontal bacteria [17]. Heparin has both direct and indirect effects on MMP/TIMP complexes circulating in blood and thereby influences matrix remodelling. Due to its antibacterial and anti-MMP activity, gallium has recently been reported as a versatile therapeutic agent in the treatment of periodontitis [18]. In this study, a systems biology approach was used to investigate the role of HBPs in periodontal disease and to identify a drug target in the treatment of periodontitis such as MMPs.

**Fig. 1:**
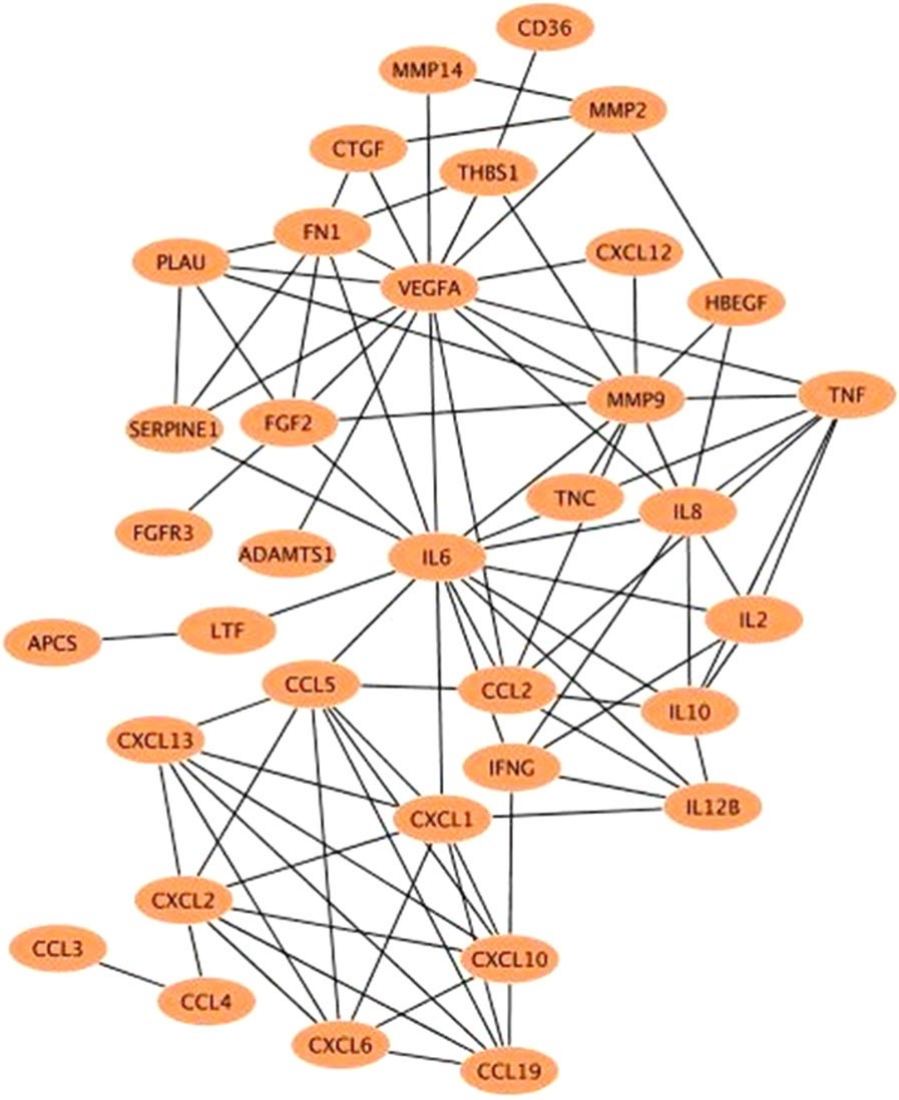
The heparin-binding putative protein interactome in periodontitis. HBPs or ‘nodes’ are *coloured* *orange* and are connected by *black lines* that denote the interactions or ‘edges’

The PubMed searches identified 249 genes that were up regulated and 146 genes that were down regulated in periodontal disease, compared with periodontal disease-free gingival samples. The HBPs among these are listed in Table 1. The list of HBPs was used to obtain interactions from STRING that was then imported into Cytoscape to build the heparin-binding putative protein interactome in periodontitis (Fig. 1). This interactome has a high clustering coefficient of 0.479 and a low number of connected components of 1 (*‘NetworkAnalyzer’*), both of which point to a high connectivity.

**Table 1:** HBPs differentially regulated in periodontal disease

Protein name	Protien abbreviation
Up regulated HBPs	
ADAM metallopeptidase with thrombospondin type 1 motif, 1	ADAMTS1
Arginase, liver	ARG1
Chemokine (C–C motif) ligand 19	CCL19
Complement factor H	CFH
Chemokine (C–X–C motif) ligand 1	CXCL1
Chemokine (C–X–C motif) ligand 12	CXCL12
Chemokine (C–X–C motif) ligand 13 (B cell chemoattractant)	CXCL13
Chemokine (C–X–C motif) ligand 2	CXCL2
Chemokine (C–X–C motif) ligand 6 (granulocyte chemotactic protein 2)	CXCL6
Fibronectin 1	FN1
Heparin-binding EGF-like growth factor	HBEGF
Insulin-like growth factor 2 mRNA binding protein 3	IGFBP6
Interleukin *10*	IL10
Interleukin *12B* (natural killer cell stimulatory factor 2, cytotoxic lymphocyte maturation factor 2, p40)	IL12B
Interleukin 6 (interferon, beta 2)	IL6
Interleukin 8	IL8
Inhibin, beta A (activin A, activin AB alpha polypeptide)	INHBA
Lactotransferrin	LTF
Matrix metallopeptidase 2 (gelatinase A, 72 kDa gelatinase, 72 kDa type IV collagenase)	MMP2
Matrix metallopeptidase 9 (gelatinase B, 92 kDa gelatinase, 92 kDa type IV collagenase)	MMP9
Plasminogen activator, urokinase	PLAU
Serpine peptidase inhibitor, clade E (nexin, plasminogen activator inhibitor type 1), member 1	SERPINE1
Tenascin C (hexabrachion)	TNC
Tumour necrosis factor	TNF
Vascular endothelial growth factor	VEGFA
Down regulated HBPs	
Amyloid P component, serum	APCS
Chemokine (C–C motif) ligand 2	CCL2
Chemokine (C–C motif) ligand 3	CCL3
Chemokine (C–C motif) ligand 4	CCL4
Chemokine (C–C motif) ligand 5	CCL5
CD36 molecule	CD36
Complement factor B	CFB
Connective tissue growth factor	CTGF
Chemokine (C–X–C motif) ligand 10	CXCL10
Fibroblast growth factor 2 (basic)	FGF2
Fibroblast growth factor receptor 3	FGFR3
Fibronectin	FN1
Glycoprotein (transmembrane)	GPNMB
Interferon, gamma	IFNG
Insulin-like growth factor binding protein 2, 36 kDa	IGFBP2
Interleukin 2	IL2
Interleukin 6 (interferon, beta 2)	IL6
Interleukin 8	IL8
Matrix metallopeptidase 14 (membrane-inserted)	MMP14
Urokinase	PLAU
Thyroglobulin	TG
Thrombospondin 1	THBS1
Tumour necrosis factor	TNF

Analyses using published data sets show 25 up-regulated and 23 down-regulated HBPs in periodontitis

Our *in silico* analyses indicate that HBPs play important roles in periodontitis. GO term enrichment analyses using DAVID show that HBPs are pivotal in cytokine activity, chemokine activity, chemokine receptor binding, growth factor activity and endopeptidase activity (Table 1; Additional file 1: Table S1). The up regulation of IL-8 reaffirms the findings of the presence of IL-8 in gingival crevicular fluid in periodontitis [19]. The cytokine IL-6 increases in expression amongst refractory periodontitis patients [20] and plays a role in bone resorption, since it stimulates the differentiation of osteoclasts and inhibits bone formation [21]. Gallium has been shown to inhibit the production of inflammatory cytokines [22] and, therefore, may have potential beneficial effect on cytokine regulation in periodontitis.

### Functional analysis of HBPs in periodontitis

In a functional analysis using DAVID, it was found that HBPs enrich a number of important biological processes such as ‘response to wounding’, ‘chemotaxis’, ‘inflammatory response’ and molecular functions such as cytokine and chemokine activity (Additional file 1: Table S1). All of these clearly underlie periodontitis and highlight the likely importance of HBPs collectively in the disease. HBPs, by virtue of their extracellular location and key functions in cell communication are readily accessible significant therapeutic targets [3–5]. Therefore, we sought to identify potential drug targets within the putative HBP interactome. For this, ‘*Cluster ONE*’ was used in Cytoscape, since it identifies HBPs which have an increased cohesiveness as a group, which is in keeping with the notion of using a systems biology approach to developing more holistic (in molecular terms) therapies.

It was reported that, during the development of periodontal disease the Lipopolysaccharides (LPS) derived from bacterial membrane have the capacity to activate host epithelial cells to express and release pro-inflammatory cytokines such as IL‐1, IL‐8, tumour necrosis factor (TNF‐α), prostaglandins and proteases [23]. The synthesis and expression of these mediators occur in a transitory and strictly controlled way under intracellular signalling pathways, which contribute to the intricacies of the inflammatory network established during the disease progression. Major signalling pathways in periodontitis comprise of the mitogen activated protein kinase (MAPK), nuclear factor kappa B (NF-κB) and janus tyrosine kinase-signal transducer and activator of the transcription (JAK/STAT) pathways [24]. Of these pathways; MAPK pathway is activated by mitogens, growth factors, stress inducers and pro-inflammatory cytokines. The results showed that the potential MAPK pathway activating HBPs were both up regulated (ADAMTS1, CXCL12, FN1, HBEGF, IL6, IL8, IL10, IL12B, MMP2, MMP9, PLAU, TNF, VFGFA,) and down regulated (CCL2, FGF2, FGFR3, FN1, IL2, IL6, IL8, MMP14 and TNF) indicating the role of HBPs (Table 1) in disease progression and homeostasis. Activation of the NF-κB pathway occurs in the presence of many pro-inflammatory mediators present in large quantities in tissues with periodontal disease such as bacterial LPS, TNF-α, IL-1, MMPs, COX2 and inducible nitric oxide synthase (iNOS) [25]. In our analyses HBPs such as TNF and MMPs were both up regulated and down regulated indicating its role in NF-κB pathway. It was reported that the JAK-STAT pathway is the signalling target of many cytokines which are thought to have biologically significant roles in periodontal disease (IFN-γ, TNF-α, IL-1 IL-4, IL-6, and IL-10) [26]. In our analyses, HBPs such as IFN-γ was down regulated suggesting that it was not involved in the activation of the JAK-STAT pathway. However, TNF-α and IL-6 was both down regulated and up regulated which indicate that the results cannot categorically establish any single target or pathways for the disease progression. It is more likely that complex interplay of different pathways take place during the disease process and pathways might be switched on and off in order to achieve homeostasis.

**Fig. 2:**
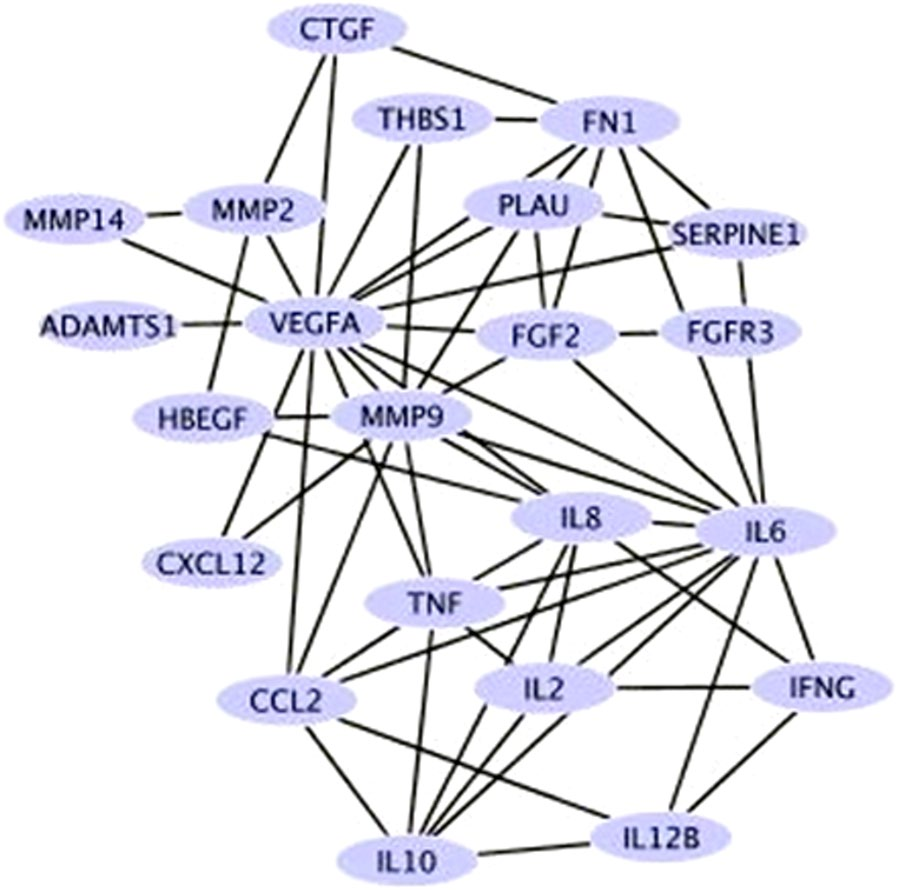
Top cluster within the putative HBP interactome. MMPs are important constituents of the top cluster of HBPs, using the *‘Cluster ONE’* plugin with Cytoscape

It was also reported when IL-6 is not present, other cytokines such as IL-1 and TNF-α induce bone resorption [27]. Both IL-6 and TNF-α were present in the top cluster within the putative HBP interactome in this study (Fig. 2). Further studies about the relationship between periodontal disease development and the cytokine network in the HBP interactome must be performed to establish the exact role of each cytokine in the inflammatory process. Chemokines, such as CXCL12, controls protection against periodontal disease associated bacteria, such as *P. gingivalis*, in normal gingival tissue and remodelling periodontal tissues to induce the production of VEGF [28]. The presence of both CXCL12 and VEGF in the top cluster within the putative HBP interactome (Fig. 2) thus suggests the important role it may have in periodontal disease homeostasis and indicate as potential drug targets. It is clear from the analyses that the major signalling pathways in periodontitis are common to various inflammatory mediators and hence their blockade may be more effective than targeting specific cytokines. However, while designing drug targets, the fact that these pathways are important in several other physiological processes and therefore their inhibition can also result in undesirable side effects should also be taken into account.

The analyses also identified MMP-2, MMP-9 and MMP-14 as being constituents within the top cluster of the putative HBP interactome (Fig. 2), a result consistent with the known roles of MMPs in matrix reorganisation and periodontium degradation. However, the membrane bound proteinase, MMP-14, found to be down-regulated in gingival tissues from periodontitis sites. Hence its role in tissue homeostasis during periodontitis still remains ambiguous. While designing targeted drug for periodontitis treatment it is important to take account of the fact that the mRNA expression data was used to predict the HBP interactome in this study. It is known that some genes could display no change in the protein expression even though changes were observed during predicted gene-interaction network. HBPs such as azurocidin which have been indicated to possess an inhibitory role in osteoclast differentiation (and thus a protective role in alveolar bone loss during the early stages of periodontitis [6]) were not identified in our searches.

The network analysis indicates that MMPs are important players in the putative HBP interactome and is an important constituent of the top cluster within the putative HBP interactome (Fig. 2). Fibroblasts secrete collagenase matrixmetalloproteinases (MMPs) causing periodontium degradation and the most common type of MMPs related to tissue destruction belongs to collagenases family (largely MMP-8 and MMP-13) with major contribution from MMP-9 and MMP-14 [29, 30]. However, in the present study it was found that MMP-14 was down-regulated in gingival tissues from periodontitis sites and therefore suggests that the use of broad spectrum anti-MMP agents should be carefully formulated to target specific HBPs associated with periodontitis. Several therapeutics has been reported to block MMPs function [31]. These includes Hydroxamate-based MMPIs (e.g. Batimastat, Marimastat and Prinomastat), Non-Hydroxamate-based MMPIs (e.g. Rebimastat, Tanomastat and Doxycycline) [31]. However, clinical successes were limited due to severe toxicities and prolonged treatment contributing to inflammation. Furthermore some of this therapeutics possesses cancer promoting activities [e.g. Batimastat promote liver metastasis, 32] and raises the concern whether designing drugs against MMPs will be beneficial. Although it was reported that MMP-1, -2, -3, -7, -8 and -9 are associated with severity of periodontitis [33], *in silico* analysis in this study only revealed MMP-2 and -9 as a major target for regulating periodontitis. Therefore, further work on elucidating the role of each MMPs in tissue homeostasis should be addressed prior to devising a new anti-MMP strategy. Recently, developing antibody based therapies to block MMPs has shown that the antibody approach was successful in blocking MMP14 function [31] but contradicted results obtained in this study indicating further analyses is required to establish its potential application in regulating periodontitis. In addition, further work should be considered as a means of activating genes down regulated in the periodontitis, such as MMP14 found in this study that may hinder the pathogenesis and thus can also form a potential drug target.

## Conclusions

In conclusion, this study attempted to integrate and rationalise available data on HBPs to identify drug targets that may play important roles in periodontitis. *In silico* analysis demonstrates that HBPs may have a role in periodontal disease and can be used for identifying potential drug targets that include chemokines, CXCL12, and proteases, MMP-2 and -9, for regulating periodontitis. The complex interactions HBPs displayed in the analyses suggest the importance of a multi-targeted approach in periodontitis treatment. From this standpoint, antibacterial and anti-MMP action of materials containing ‘gallium’, which are also reported to inhibit the production of inflammatory cytokines, are promising candidates for its potential application in periodontitis treatment.

## Availability of supporting data section

All the supporting data are included as additional files. Online database resources used in this study:

STRING v9.1. doi: 10.1093/nar/gks1094.http://string.embl.de/

Cytoscape 2.8. doi: 10.1093/bioinformatics/btq675. http://www.cytoscape.org/download_old_versions.html

DAVID bioinformatics resources. doi: 10.1038/nprot.2008.211. https://david.ncifcrf.gov/content.jsp?file=release.html.

## Additional file


10.1186/s13104-016-1857-1 Supplementary material which encompasses; Top gene ontology (GO) terms (biological process; BP and molecular function; MF) enriched to periodontitis heparin-binding protein (HBP) dataset. The lists of 249 genes that were up regulated and 146 genes that were down regulated in periodontal disease, compared with periodontal disease-free gingival samples are presented with corresponding references.

## Abbreviations

HBP: heparin-binding protein
MMP: matrixmetalloproteinase
TIMP: tissue inhibitor of metalloproteinase
STRING: Search Tool for the Retrieval of Interacting Genes
GO: gene ontology
BP: biological process
CC: cellular component
MF: molecular function
DAVID: Database for annotation, visualization and integrated discovery
IL: interleukin
