# Supplementary material for: Insilico analyses of heparin binding proteins expression in human periodontal tissues
Source: BMC Res Notes. 2016 Jan 28;9:53. doi: 10.1186/s13104-016-1857-1 (PMC4731997; doi:10.1186/s13104-016-1857-1)
Supplement: Supplementary file 1 — 10.1186/s13104-016-1857-1 Supplementary material which encompasses; Top gene ontology (GO) terms (biological process; BP and molecular function; MF) enriched to periodontitis heparin-binding protein (HBP) dataset. The lists of 249 genes that were up regulated and 146 genes that were down regulated in periodontal disease, compared with periodontal disease-free gingival samples are presented with corresponding references. [file 13104_2016_1857_MOESM1_ESM.docx]

**Supplementary material**

**Analyses of heparin binding proteins expression in periodontal tissues**

Bernadette Lackey^1^, Quentin M. Nunes^2^, Susan M Higham^1^, David G Fernig^3^, Sabeel P Valappil^1*^

*1) Department of Health Services Research and School of Dentistry, University of Liverpool, Research Wing, Daulby Street, Liverpool, L69 3GN, United Kingdom*

*2) NIHR Liverpool Pancreas Biomedical Research Unit, Royal Liverpool University Hospital, Daulby Street, Liverpool L69 3GA, United Kingdom*

*3) Department of Structural and Chemical Biology, Institute of Integrative Biology, University of Liverpool, Crown Street, Liverpool, L69 7ZB, United Kingdom*

Short title: HBP and periodontitis

Key words: periodontitis; heparin, heparan sulfate,

*****Corresponding Author. Mailing address: Department of Health Services Research and School of Dentistry, University of Liverpool, Research Wing, Daulby Street, Liverpool, L69 3GN, UK Tel +44 (0)151 706 5299, Fax +44 (0)151 706 5809 Email: [S.Valappil@liv.ac.uk](mailto:S.Valappil@liv.ac.uk)

**Table 1.** Top GO terms (BP and MF) enriched to periodontitis HBP dataset.

| TOP GO TERMS (BP) ENRICHED TO PERIODONTITIS HBP DATASET | | | | | | | | | | | | | | | | | | | | | | | |
| --- | --- | --- | --- | --- | --- | --- | --- | --- | --- | --- | --- | --- | --- | --- | --- | --- | --- | --- | --- | --- | --- | --- | --- |
|  | | Term | | | Count | | | | % | | | | | PValue | | | Genes | | | | | | |
| GOTERM_BP_FAT | | GO:0009611~response to wounding | | | 26 | | 60.47 | | | | | 5.13E-25 | | | | | CXCL1, CCL3, TNF, CCL2, TNC, CXCL2, CXCL6, CCL5, CCL4, IL10, CXCL10, CTGF, SERPINE1, CFH, THBS1, FGF2, FN1, IL6, APCS, IL8, CFB, CCL19, CD36, CXCL13, HBEGF, PLAU | | | | | | |
| GOTERM_BP_FAT | | GO:0006935~chemotaxis | | | 17 | | 39.53 | | | | | 8.58E-21 | | | | | CXCL1, CCL3, IL6, CCL2, IL8, CXCL2, CCL19, CXCL6, CCL5, CXCL12, CCL4, IL10, CXCL10, CXCL13, IFNG, FGF2, PLAU | | | | | | |
| GOTERM_BP_FAT | | GO:0042330~taxis | | | 17 | | 39.53 | | | | | 8.58E-21 | | | | | CXCL1, CCL3, IL6, CCL2, IL8, CXCL2, CCL19, CXCL6, CCL5, CXCL12, CCL4, IL10, CXCL10, CXCL13, IFNG, FGF2, PLAU | | | | | | |
| GOTERM_BP_FAT | | GO:0006954~inflammatory response | | | 19 | | 44.19 | | | | | 9.06E-19 | | | | | CXCL1, IL6, CCL3, TNF, CCL2, APCS, IL8, CFB, CXCL2, CCL19, CXCL6, CCL5, CCL4, IL10, CXCL10, CXCL13, CFH, THBS1, FN1 | | | | | | |
| GOTERM_BP_FAT | | GO:0006955~immune response | | | 23 | | 53.49 | | | | | 4.99E-18 | | | | | CXCL1, IL6, CCL3, TNF, CCL2, IL8, CFB, CXCL2, CCL19, CXCL6, CCL5, CXCL12, CCL4, IL10, CXCL10, CXCL13, VEGFA, IFNG, CFH, LTF, IL12B, THBS1, IL2 | | | | | | |
| GOTERM_BP_FAT | | GO:0006952~defense response | | | 22 | | 51.16 | | | | | 9.72E-18 | | | | | CXCL1, IL6, CCL3, TNF, CCL2, APCS, IL8, CFB, CXCL2, CCL19, CXCL6, CCL5, CCL4, IL10, CXCL10, INHBA, CXCL13, IFNG, CFH, LTF, THBS1, FN1 | | | | | | |
| GOTERM_BP_FAT | | GO:0007626~locomotory behavior | | | 17 | | 39.53 | | | | | 5.22E-17 | | | | | CXCL1, CCL3, IL6, CCL2, IL8, CXCL2, CCL19, CXCL6, CCL5, CXCL12, CCL4, IL10, CXCL10, CXCL13, IFNG, FGF2, PLAU | | | | | | |
| GOTERM_BP_FAT | | GO:0016477~cell migration | | | 16 | | 37.21 | | | | | 1.87E-15 | | | | | IL6, TNF, CCL2, IL8, MMP14, CCL5, CXCL12, IL10, CTGF, IFNG, HBEGF, IL12B, THBS1, FGF2, PLAU, FN1 | | | | | | |
| GOTERM_BP_FAT | | GO:0051674~localization of cell | | | 16 | | 37.21 | | | | | 8.82E-15 | | | | | IL6, TNF, CCL2, IL8, MMP14, CCL5, CXCL12, IL10, CTGF, IFNG, HBEGF, IL12B, THBS1, FGF2, PLAU, FN1 | | | | | | |
| GOTERM_BP_FAT | | GO:0048870~cell motility | | | 16 | | 37.21 | | | | | 8.82E-15 | | | | | IL6, TNF, CCL2, IL8, MMP14, CCL5, CXCL12, IL10, CTGF, IFNG, HBEGF, IL12B, THBS1, FGF2, PLAU, FN1 | | | | | | |
|  | | |  | | |  | | | | |  | | | | |  | | |  | | | | |
| TOP GO TERMS (MF) ENRICHED TO PERIODONTITIS HBP DATASET | | | | | | | | | | | | | | | | | | | | | | | |
|  | | Term | | | | Count | | % | | | PValue | | | | | Genes | | | | | | | |
| GOTERM_MF_FAT | | GO:0005125~cytokine activity | | | 20 | | | 46.51 | | | 9.17E-25 | | | | | CXCL1, CCL3, IL6, TNF, CCL2, IL8, CXCL2, CCL19, CXCL6, CCL5, CXCL12, CCL4, IL10, CXCL10, INHBA, CXCL13, VEGFA, IFNG, IL12B, IL2 | | | | | | | |
| GOTERM_MF_FAT | | GO:0008009~chemokine activity | | | 12 | | | 27.91 | | | 6.47E-19 | | | | | CXCL1, CCL3, CCL2, IL8, CXCL13, CXCL2, CCL19, CXCL6, CCL5, CCL4, CXCL12, CXCL10 | | | | | | | |
| GOTERM_MF_FAT | | GO:0042379~chemokine receptor binding | | | 12 | | | 27.91 | | | 1.40E-18 | | | | | CXCL1, CCL3, CCL2, IL8, CXCL13, CXCL2, CCL19, CXCL6, CCL5, CCL4, CXCL12, CXCL10 | | | | | | | |
| GOTERM_MF_FAT | | GO:0008201~heparin binding | | | 10 | | | 23.26 | | | 1.95E-11 | | | | | CCL2, CTGF, VEGFA, HBEGF, ADAMTS1, CXCL6, GPNMB, THBS1, FGF2, FN1 | | | | | | | |
| GOTERM_MF_FAT | | GO:0008083~growth factor activity | | | 11 | | | 25.58 | | | 4.00E-11 | | | | | CXCL1, INHBA, IL6, CTGF, VEGFA, HBEGF, IL12B, FGF2, CXCL12, IL10, IL2 | | | | | | | |
| GOTERM_MF_FAT | | GO:0005539~glycosaminoglycan binding | | | | 10 | | 23.26 | | | 3.13E-10 | | | | | CCL2, CTGF, VEGFA, HBEGF, ADAMTS1, CXCL6, GPNMB, THBS1, FGF2, FN1 | | | | | | | |
| GOTERM_MF_FAT | | GO:0001871~pattern binding | | | | 10 | | 23.26 | | | 7.34E-10 | | | | | CCL2, CTGF, VEGFA, HBEGF, ADAMTS1, CXCL6, GPNMB, THBS1, FGF2, FN1 | | | | | | | |
| GOTERM_MF_FAT | | GO:0030247~polysaccharide binding | | | | 10 | | 23.26 | | | 7.34E-10 | | | | | CCL2, CTGF, VEGFA, HBEGF, ADAMTS1, CXCL6, GPNMB, THBS1, FGF2, FN1 | | | | | | | |
| GOTERM_MF_FAT | | GO:0030246~carbohydrate binding | | | | 12 | | 27.91 | | | 6.04E-09 | | | | | APCS, CCL2, CTGF, VEGFA, HBEGF, ADAMTS1, CXCL6, GPNMB, THBS1, FGF2, IL2, FN1 | | | | | | | |
| GOTERM_MF_FAT | | GO:0004175~endopeptidase activity | | | | 8 | | 18.60 | | | 1.30E-04 | | | | | CFB, MMP9, SERPINE1, LTF, ADAMTS1, MMP14, MMP2, PLAU | | | | | | | |
| TOP GO TERMS (BP) ENRICHED TO PERIODONTITIS HBP DATASET | | | | | | | | | | | | | | | | | | |  |  |  |  |  |
|  | | | Term | | | | | | List Total | | | Pop Hits | | Pop Total | | | Fold Enrichment | | Bonferroni | Benjamini | FDR |  |  |
| GOTERM_BP_FAT | | | GO:0009611~response to wounding | | | | | | 43 | | | 530 | | 13528 | | | 15.43 | | 6.17E-22 | 6.17E-22 | 8.30E-22 |  |  |
| GOTERM_BP_FAT | | | GO:0006935~chemotaxis | | | | | | 43 | | | 160 | | 13528 | | | 33.43 | | 1.03E-17 | 5.16E-18 | 1.39E-17 |  |  |
| GOTERM_BP_FAT | | | GO:0042330~taxis | | | | | | 43 | | | 160 | | 13528 | | | 33.43 | | 1.03E-17 | 5.16E-18 | 1.39E-17 |  |  |
| GOTERM_BP_FAT | | | GO:0006954~inflammatory response | | | | | | 43 | | | 325 | | 13528 | | | 18.39 | | 1.09E-15 | 3.63E-16 | 1.47E-15 |  |  |
| GOTERM_BP_FAT | | | GO:0006955~immune response | | | | | | 43 | | | 690 | | 13528 | | | 10.49 | | 6.00E-15 | 1.50E-15 | 8.07E-15 |  |  |
| GOTERM_BP_FAT | | | GO:0006952~defense response | | | | | | 43 | | | 615 | | 13528 | | | 11.25 | | 1.17E-14 | 2.34E-15 | 1.57E-14 |  |  |
| GOTERM_BP_FAT | | | GO:0007626~locomotory behavior | | | | | | 43 | | | 274 | | 13528 | | | 19.52 | | 6.28E-14 | 1.05E-14 | 8.44E-14 |  |  |
| GOTERM_BP_FAT | | | GO:0016477~cell migration | | | | | | 43 | | | 276 | | 13528 | | | 18.24 | | 2.27E-12 | 3.24E-13 | 3.05E-12 |  |  |
| GOTERM_BP_FAT | | | GO:0051674~localization of cell | | | | | | 43 | | | 307 | | 13528 | | | 16.40 | | 1.05E-11 | 1.32E-12 | 1.42E-11 |  |  |
| GOTERM_BP_FAT | | | GO:0048870~cell motility | | | | | | 43 | | | 307 | | 13528 | | | 16.40 | | 1.05E-11 | 1.32E-12 | 1.42E-11 |  |  |
|  | | |  | | | | | |  | | |  | |  | | |  | |  |  |  |  |  |
| TOP GO TERMS (MF) ENRICHED TO PERIODONTITIS HBP DATASET | | | | | | | | | | | | | | | | | | |  |  |  |  |  |
|  | | | Term | | | | | | List Total | | | Pop Hits | | Pop Total | | | Fold Enrichment | | Bonferroni | Benjamini | FDR |  |  |
| GOTERM_MF_FAT | | | GO:0005125~cytokine activity | | | | | | 41 | | | 195 | | 12983 | | | 32.48 | | 1.19E-22 | 1.19E-22 | 1.07E-21 |  |  |
| GOTERM_MF_FAT | | | GO:0008009~chemokine activity | | | | | | 41 | | | 46 | | 12983 | | | 82.61 | | 8.41E-17 | 4.21E-17 | 7.54E-16 |  |  |
| GOTERM_MF_FAT | | | GO:0042379~chemokine receptor binding | | | | | | 41 | | | 49 | | 12983 | | | 77.55 | | 1.82E-16 | 6.08E-17 | 1.63E-15 |  |  |
| GOTERM_MF_FAT | | | GO:0008201~heparin binding | | | | | | 41 | | | 103 | | 12983 | | | 30.74 | | 2.53E-09 | 6.32E-10 | 2.27E-08 |  |  |
| GOTERM_MF_FAT | | | GO:0008083~growth factor activity | | | | | | 41 | | | 161 | | 12983 | | | 21.64 | | 5.20E-09 | 1.04E-09 | 4.66E-08 |  |  |
| GOTERM_MF_FAT | | | GO:0005539~glycosaminoglycan binding | | | | | | 41 | | | 140 | | 12983 | | | 22.62 | | 4.07E-08 | 6.79E-09 | 3.65E-07 |  |  |
| GOTERM_MF_FAT | | | GO:0001871~pattern binding | | | | | | 41 | | | 154 | | 12983 | | | 20.56 | | 9.55E-08 | 1.36E-08 | 8.55E-07 |  |  |
| GOTERM_MF_FAT | | | GO:0030247~polysaccharide binding | | | | | | 41 | | | 154 | | 12983 | | | 20.56 | | 9.55E-08 | 1.36E-08 | 8.55E-07 |  |  |
| GOTERM_MF_FAT | | | GO:0030246~carbohydrate binding | | | | | | 41 | | | 354 | | 12983 | | | 10.73 | | 7.85E-07 | 9.81E-08 | 7.03E-06 |  |  |
| GOTERM_MF_FAT | | | GO:0004175~endopeptidase activity | | | | | | 41 | | | 375 | | 12983 | | | 6.76 | | 0.0167349 | 0.001873 | 0.151066 |  |  |

**Table 2.** List of up-regulated genes in periodontitis with corresponding references.

| Symbol | Gene Name | | Source (Paper) |
| --- | --- | --- | --- |
| ADAM8 | A disintegrin and metalloproteinase domain 8 | Milward *et al.* 2007 | |
| ADAMTS1 | ADAM metallopeptidase with thrombospondin type 1 motif, 1 | Beikler *et al.* 2008 | |
| ADFP | Adipose differentiation-related protein | Wright *et al.* 2011 | |
| AKAP12 | A kinase (PRKA) anchor protein (gravin) 12 | Milward *et al.* 2007 | |
| AREG | Amphiregulin (schwannoma-derived growth factor) | Milward *et al.* 2007 | |
| ARG1 | Arginase, liver | Jönsson *et al.* 2011 | |
| ARG2 | Arginase, type II | Milward *et al.* 2007 | |
| ARHGAP8 | Homo sapiens rho GTPASE activating protein 8 | Kim *et al.* 2006 | |
| ARL4A | ADP-ribosylation factor-like 4A | Wright *et al.* 2011 | |
| ATP6V0A4 | ATPase, H+ transporting, lysosomal V0 subunit a4 | Jönsson *et al.* 2011 | |
| BIRC1 | Baculoviral IAP repeat-containing 1 | Beikler *et al.* 2008 | |
| BIRC3 | Baculoviral IAP repeat-containing 3 | Milward *et al.* 2007 | |
| BTG2 | BTG family, member 2 | Wright *et al.* 2011 | |
| C-JUN | jun proto-oncogene | Beikler *et al.* 2008 | |
| C-Kit | KIT v-kit Hardy-Zuckerman 4 feline sarcoma viral oncogene homolog (*Homo sapiens*) | Beikler *et al.* 2008 | |
| C14orf111 | Chromosome 14 opend reading frame 111 | Wright *et al.* 2011 | |
| C15orf48 | Chromosome 15 open reading frame 48 | Jönsson *et al.* 2011 | |
| C16orf74 | Chromosome 16 open reading frame 74 | Jönsson *et al.* 2011 | |
| CASP-10 | Caspase-10 | Kim *et al.* 2006 | |
| CCL19 | Chemokine (C-C motif) ligand 19 | Jönsson *et al.* 2011 | |
| CCL20 | Chemokine (C-C motif) ligand 20 | Milward *et al.* 2007 | |
| CCND2 | Cyclin D2 | Milward *et al.* 2007 | |
| CCR3 | Chemokine (C-C motif) receptor 3 | Beikler *et al.* 2008 | |
| CD14 | CD14 molecule | Wang *et al.* 2003 | |
| CD15 | 3-fuctosyl-N-acetyl-lactosamine | Beikler *et al.* 2008 | |
| CD177 | CD177 molecule | Jönsson *et al.* 2011 | |
| CD27 | CD27 molecule | Beikler *et al.* 2008 | |
| CD38 | CD38 Molecule | Beikler *et al.* 2008 | |
| CDKN3 | Cyclin-dependent kinase inhibitor 3 (CDK2-asociated dual specificity phosphatase) | Jönsson *et al.* 2011 | |
| CEBPD | CCAAT/enhancer binding protein (C/EBP), delta | Beikler *et al.* 2008 | |
| CFH | Complement factor H | Abe *et al.*2011. | |
| CGI-14 | CGI- 14 protein | Wright *et al.* 2011 | |
| CH25H | Cholesterol 25-hydroxylase | Beikler *et al.* 2008 | |
| CKS2 | CDC28 protein kinase regulatory subunit 2 | Wright *et al.* 2011 | |
| CMP | Carboxypeptidase M | Jönsson *et al.* 2011 | |
| CRISP3 | Cysteine-ruch secretory protein 3 | Jönsson *et al.* 2011 | |
| CRY1 | Cryptochromw 1 (photolyase-like) | Wright *et al.* 2011 | |
| CSF1 | Colony stimulating factor 1 (macrophage) | Wright *et al.* 2011 | |
| CTBP1 | C-terminal binding protein1 | Wright *et al.* 2011 | |
| CUTL1 | Cut-like, CCAAT displacement protein (Drosophila) | Jönsson *et al.* 2011 | |
| CXCL1 | Chemokine (C-X-C motif) ligand 1 | Abe *et al.*2011, Wright *et al.* 2011 | |
| CXCL12 | Chemokine (C-X-C motif) ligand 12 | Abe *et al.*2011. | |
| CXCL13 | Chemokine (C-X-C motif) ligand 13 (B-cell chemoattractant) | Jönsson *et al.* 2011 | |
| CXCL2 | Chemokine (C-X-C motif) ligand 2 | Milward *et al.* 2007, Wright *et al.* 2011 | |
| CXCL3 | Chemokine (C-X-C motif) ligand 3 | Beikler et al. 2008, Wright *et al.* 2011 | |
| CXCL6 | Chemokine (C-X-C motif) ligand 6 (granulocyte chemotactic protein 2) | Jönsson *et al.* 2011 | |
| CXCL9 | Chemokine (C-X-C motif) ligand 9 | Beikler *et al.* 2008 | |
| CXCR1 | Chemokine (C-X-C motif) receptor 1 | Beikler *et al.* 2008 | |
| CXCR4 | Chemokine (C-X-C motif) receptor 4 | Beikler *et al.* 2008 | |
| CXD3 | Gap junction protein, delta 3 | Beikler *et al.* 2008 | |
| DCT | Dopachrome tautomerase (dopachrome delta-isomerase, tyrosine-related protein 2) | Jönsson *et al.* 2011 | |
| DDX3Y | DEAD (Asp-Glu-Ala-Asp) box polypeptide 3, Y-linked | Wright *et al.* 2011 | |
| DNAJB1 | DnaJ (Hsp40) homologue, subfamily B, member1 | Wright *et al.* 2011 | |
| DNAJB9 | DnaJ (Hsp40) homolog, subfamily B, member 9 | Milward et al. 2007, Wright *et al.* 2011 | |
| DNCL 12 | Dyneinm cytoplasmic, light intermediate polypeptide 2 | Wright *et al.* 2011 | |
| DSC1 | Desmocollin 1 | Jönsson *et al.* 2011 | |
| DSCR1 | Down syndrome critical region gene 1 | Beikler *et al.* 2008 | |
| DUSP1 | Dual specificity phosphatase 1 | Wright *et al.* 2011 | |
| DUSP2 | Dual specificity phosphatase 2 | Beikler *et al.* 2008 | |
| DUSP6 | Dual specificity phosphatase 6 | Milward *et al.* 2007 | |
| EPHA3 | EPH receptor A3 | Abe *et al.*2011. | |
| EREG | Epiregulin | Milward *et al.* 2007 | |
| ERO1L | ERO1-like (S. cerevisiase) | Jönsson *et al.* 2011 | |
| F2RL1 | 60165928R1 NIH_MGC_70 H. *sapiens* cDNA clone | Wright *et al.* 2011 | |
| FAM46C | Family with sequence similarity 46, member C | Jönsson *et al.* 2011 | |
| FBXL14 | F-box and leucine-rich repeat protein 14 | Wright *et al.* 2011 | |
| FEZ1 | Fasciculation and elongation protein zeta 1 (zygin I) | Milward *et al.* 2007 | |
| FLJ14281 | Hypthetical protein FLJ14281 DnaJ (Hsp40) Homologue, subfamily B, member 14 | Wright *et al.* 2011 | |
| FN1 | Fibronectin 1 | Abe *et al.*2011. | |
| Fos | FBJ murine osteosarcoma viral oncogene homolog | Bodet *et al*. 2007, Wright *et al.* 2011, Beikler *et al.* 2008, Tanabe *et al.* 2008 | |
| FOSB | V-FOS FBJ murine osteosarcoma viral oncogene homolog B | Kim *et al.* 2006 | |
| FOSL1 | FOS-like antigen 1 | Milward *et al.* 2007 | |
| FUT3 | Fucosyltransferase 3 (galactoside 3(4)-L-fucosyltransferase, Lewis blood group) | Jönsson *et al.* 2011 | |
| FUT5 | Fucosyltransferase 5 | Jönsson *et al.* 2011 | |
| GADD45B | Growth arrest and DNA-damage-inducible, beta | Beikler *et al.* 2008 | |
| GDF15 | Growth differentiation factor 15 | Milward *et al.* 2007 | |
| CLCA4 | Chloride channel, calcium activated, family member 4 | Jönsson *et al.* 2011 | |
| GNPDA1 | Glucosamine-6-phosphate deaminase 1 | Wright *et al.* 2011 | |
| GPB1 | G-protein β Subunit | Beikler *et al.* 2008 | |
| GPR37 | G protein-coupled receptor 37 (endothelin receptor type B-like) | Jönsson *et al.* 2011 | |
| GPR84 | G protein-coupled receptor 84 | Beikler *et al.* 2008 | |
| GTL3 | Like; orthologue of mouse gene trap locus 3 | Wright *et al.* 2011 | |
| GYS2 | Glycogen synthase 2 (liver) | Jönsson *et al.* 2011 | |
| H1F0 | H1 hitone family, member 0 | Wright *et al.* 2011 | |
| H2BFA | *Homo sapiens* cDNA clone IMAGE:2989839, with apprent retained intron | Wright *et al.* 2011 | |
| HBA1 | Hemoglobin, alpha1 | Jönsson *et al.* 2011 | |
| HBA2 | Hemoglobin, alpha 2 | Jönsson *et al.* 2011 | |
| HBB | Hemoglobin, beta | Jönsson *et al.* 2011 | |
| HBEGF | Heparin-binding EGF-like growth factor | Milward *et al.* 2007 | |
| HERPUD1 | Homocysteine-inducible, endoplasmic reticulum stress-inducible, ubiquitin-like domain member 1 | Milward *et al.* 2007 | |
| HES1 | Hairy and enhancer of split 1, (*Drosophila*) | Wright *et al.* 2011 | |
| HHEX | Hematopoietically expressed homeobox | Beikler *et al.* 2008 | |
| HIST1H2AI | Histone 1, H2ai | Wright *et al.* 2011 | |
| HIST1H3D | Histone 1, H3d | Wright *et al.* 2011 | |
| HIST1H2BG | Histone cluster 1, H2bg | Wright *et al.* 2011 | |
| HK1 | Hexokinase 1 | Wright *et al.* 2011 | |
| HK2 | Hexokinase 2 | Milward *et al.* 2007 | |
| HLA-DQB1 | Major histocompatibility complex, class11, DQ β1 | Wright *et al.* 2011 | |
| HMGA2 | High mobility group AT-hook 2 /// high mobility group AT-hook 2 | Milward *et al.* 2007 | |
| HMOX1 | [Heme oxygenase (decycling) 1](http://www.genecards.org/cgi-bin/carddisp.pl?gene=HMOX1&search=hmox1) | Beikler *et al.* 2008, Wright *et al.* 2011 | |
| HPK1 | Hematopoietic progenitor kinase 1 | Bodet *et al*. 2007. | |
| HSPA1A | Heat shock 70kDa protein 1A | Wright *et al.* 2011 | |
| HSPA1B | Heat shock 70kDa protein 1B | Wright *et al.* 2011 | |
| HSPA6 | Unnamed protein product; heat-shock protein HSP70B | Wright *et al.* 2011 | |
| HSPA8 | Heat shock 70kDa protein 8 | Wright *et al.* 2011 | |
| HSPCA | Heat shock 90kDa protein 1, α | Wright *et al.* 2011 | |
| HSPE1 | Heat shoch 10 kDa protein 1 (chaperonin 10) | Wright *et al.* 2011 | |
| HSPH1 | Heat shock 105kDa/110 kDa protein 1 | Wright *et al.* 2011 | |
| ICAM1 | IIntercellular adhesion molecule 1 | Beikler *et al.* 2008 | |
| ID2 | Inhibitor of DNA binding 2, dominant negative helix-loo-helix protein | Wright *et al.* 2011 | |
| IFI-15 | Interferon-induced protein IFI-15k | Kim *et al.* 2006 | |
| IFRD1 | Interferon-related developmental regulator 1 | Milward *et al.* 2007 | |
| IGF2BP3 | insulin-like growth factor 2 mRNA binding protein 3 | Jönsson *et al.* 2011 | |
| IGFBP6 | Insulin-like growth factor 2 mRNA binding protein 3 | Jönsson *et al.* 2011 | |
| IGJ | Immunoglobin J polypeptide, linker protein | Abe *et al.*2011. | |
| IGSF6 | Immunoglobin supefamily, member 6 | Beikler *et al.* 2008 | |
| IL10 | Interleukin *10* | Beikler *et al.* 2008 | |
| IL11 | Interleukin *11* | Beikler *et al.* 2008 | |
| IL12A | Interleukin 12A (natural killer cell stimulatory factor 1, cytotoxic lymphocyte maturation factor 1, p35) | Beikler *et al.* 2008 | |
| IL12B | Interleukin *12B* (natural killer cell stimulatory factor 2, cytotoxic lymphocyte maturation factor 2, p40) | Beikler *et al.* 2008 | |
| IL15RA | Interleukin 15 receptor, alpha | Beikler *et al.* 2008 | |
| IL16 | Interleukin *16* | Beikler *et al.* 2008 | |
| IL18 | Interleukin *18* (interferon*-*gamma*-*inducing factor) | Beikler *et al.* 2008 | |
| IL24 | Interleukin-24 | Kim *et al.* 2006 | |
| IL2R | Interleukin 2 receptor | Beikler *et al.* 2008 | |
| IL8R | Interleukin 8 receptor | Beikler *et al.* 2008 | |
| IL1A | Interleukin alpha | Wang *et al.* 2003 | |
| IL1A | Interleukin 1, alpha | Milward *et al.* 2007 | |
| IL1B | Interleukin 1, beta | Milward *et al.* 2007 | |
| IL1F9 | Interleukin 1 family, member 9 | Milward *et al*. 2007 | |
| IL32 | Interleukin 32 | Milward *et al.* 2007 | |
| IL6 | interleukin 6 (interferon, beta 2) | Tanabe *et al*. 2008, Wang *et al.* 2003 | |
| IL6R | Interleukin 6 receptor | Jönsson *et al.* 2011 | |
| IL8 | interleukin 8 | Tanabe *et al.* 2008, Milward *et al.* 2007, Wang *et al.* 2003 | |
| IL 1B | Interleukin 1beta | Abe *et al.*2011, Wang *et al.* 2003 | |
| INHBA | Inhibin, beta A (activin A, activin AB alpha polypeptide) | Milward *et al.* 2007 | |
| LTA4H | Leukotriene A4 hydrolase | Beikler *et al.* 2008 | |
| ITGB8 | Integrin, beta 8 | Beikler *et al.* 2008 | |
| JNK | Mitogen-activated protein kinase 8 | Bodet *et al*. 2007. | |
| Jun | *jun* proto-oncogene | Bodet *et al*. 2007. | |
| KLF13 | Kruppel-like factor 13 | Jönsson *et al.* 2011 | |
| LCP2 | Lymphocyte cystolic protein 2 | Abe *et al.*2011. | |
| LTF | Lactotransferrin | Kim *et al.* 2006 | |
| MAFF | v-maf musculoaponeurotic fibrosarcoma oncogene homolog F (avian) | Milward *et al.* 2007, Wright *et al.* 2011 | |
| MAFG | v-maf musculoaponeurotic fibrosarcoma oncogene homologue G (avian) | Wright *et al.* 2011 | |
| MCP1 | Monocyte chemotactic protein-1 (Chemokine ligand 2) | Tanabe *et al*. 2008. | |
| MGC23985 | Similar to AVLV472 | Jönsson *et al.* 2011 | |
| MGC31930 | Colony-stimulating factor (CSF-1) precursor; Human macrophage-specfic colony stimulating factor (CSF-1) Mrnac, CSoFm1p; 1MetCe ScFd;s | Wright *et al.* 2011 | |
| MGC5566 | Homo sapiens hypothetical protein MGC5566 | Kim *et al.* 2006 | |
| MGP | Matrix Gla protein | Abe *et al.*2011. | |
| MIAL | Otoraplin | Beikler *et al.* 2008 | |
| MICAL2 | Microtubule associated monoxygenase, calponin and LIM domain containing 2 | Milward *et al.* 2007 | |
| MKK1 | Mitogen-activated protein kinase kinase 1 | Tanabe *et al*. 2008. | |
| MKK2 | Mitogen-activated protein kinase kinase 2 | Tanabe *et al*. 2008. | |
| MKK3 | Mitogen-activated protein kinase 3 | Bodet *et al*. 2007. | |
| MKK6 | Mitogen-activated protein kinase kinase 6 | Tanabe *et al*. 2008. | |
| MKK6 | Mitogen-activated protein kinase 6 | Bodet *et al*. 2007. | |
| MMD | Monocyte to macrophage differentiation-associated | Milward *et al.* 2007 | |
| MMP1 | Matrix metalloproteinase-1 | Kim *et al.* 2006, Beikler *et al.* 2003 | |
| MMP9 | Matrix metallopeptidase 9 (gelatinase B, 92kDa gelatinase, 92kDa type IV collagenase) | Beikler *et al.* 2008 | |
| MMP2 | matrix metallopeptidase 2 (gelatinase A, 72kDa gelatinase, 72kDa type IV collagenase) | Bodet *et al*. 2007. | |
| MMP3 | Matrix metallopeptidase 3 (stromelysin 1, progelatinase | Bodet *et al*. 2007, Tanabe *et al.* 2008, Kim *et al*. 2006 | |
| MS4A1 | Membrane-spanning 4-domains, subfamily A, member 1 | Abe *et al.*2011, Jönsson *et al.* 2011 | |
| MSK1 | Mitigen- and stress-activated protein kinase 1 | Bodet *et al*. 2007. | |
| MT1H | Metallothionein 1H | Wright *et al.* 2011 | |
| MT2A | Metallothionein 12A | Wright *et al.* 2011 | |
| MT1L | Metallothionein IL | Wright *et al.* 2011 | |
| MTIX | Metallothionein 1X | Wright *et al.* 2011 | |
| MX1 | [Myxovirus (influenza virus) resistance 1, interferon-inducible protein p78 (mouse)](http://www.genecards.org/cgi-bin/carddisp.pl?gene=MX1&search=mx1) | Beikler *et al.* 2008 | |
| NEK1 | NIMA (never in mitosis gene a)-related kinase 1 | Jönsson *et al.* 2011 | |
| NFkB p50 | NF-kappa-B p50 | Bodet *et al*. 2007, Tanabe *et al.* 2008 | |
| NFkB p65 | NF-kappa-B p65 | Bodet *et al*. 2007, Tanabe *et al*. 2008 | |
| NR4A1 | Nuclear receptor subfamily4, group A, member 1 | Wright *et al.* 2011 | |
| NR4A2 | Nuclear receptor subfamily 4, group A, member 2 | Wright *et al.* 2011 | |
| NSFL1C | NSFL1 (p9) cofactor (p47) | Wright *et al.* 2011 | |
| ODC1 | Ornithine decarboxylase 1 | Wright *et al.* 2011 | |
| OMD | Osteomodulin | Abe *et al.*2011. | |
| ORM1 | Orosomucoid 1 | Wright *et al.* 2011 | |
| OSM | Oncostatin M | Wright *et al.* 2011 | |
| Osteoglycin | Osteoglycin | Abe *et al.*2011. | |
| p38 MAPK | p38 Mitogen-activated protein kinase | Bodet *et al*. 2007. | |
| PAPSS2 | 3'-phosphoadenosine 5'-phosphosulfate synthase 2 | Jönsson *et al.* 2011 | |
| PDE4B | Phosphodiesterase 4B, cAMP-specific | Beikler *et al.* 2008 | |
| PDGFD | Platelet-derived growth factor D | Abe *et al.*2011. | |
| PDZRN4 | PDZ domain containing RING finger 4 | Jönsson *et al.* 2011 | |
| PFKFB3 | 6-Phosphofructo-2-kinase/fructose-2,6-biphosphatase 3 | Wright *et al.* 2011 | |
| PGM3 | Phosphoglucomutase 3 | Milward *et al.* 2007 | |
| PHLDA1 | Pleckstrin homology-like domain, family A, member 1 | Milward *et al.* 2007, Jönsson *et al.* 2011 | |
| PHLDA2 | Plecktrin homology-like domainm family A, member 2 | Wright *et al.* 2011 | |
| PLAU | Plasminogen activator, Urokinase | Milward *et al.* 2007 | |
| PLAUR | Plasminogen activator, urokinase receptor | Milward *et al.* 2007 | |
| POU2AF1 | POU domain, class 2, associating factor 1 | Jönsson *et al.* 2011 | |
| PPAP2B | Phosphatidic acid phosphatase type 2B | Wright *et al.* 2011 | |
| PPP1R3C | Protein phosphatase 1, regulatory (inhibitor) subunit 3C | Jönsson *et al.* 2011 | |
| PRG1 | p53-responsive gene 1 | Beikler *et al.* 2008 | |
| PTDSR | Phophatidylserine receptor | Wright *et al.* 2011 | |
| PTP4A1 | 60213508F1 NIH_MGC_81 H. *sapiens* cDNA clone | Wright *et al.* 2011 | |
| PTX3 | Pentraxin 3, long | Beikler *et al.* 2008 | |
| PYGL | Phosphorylase, glycogen; liver (Hers disease, glycogen storage disease type VI) | Jönsson *et al.* 2011 | |
| QSER1 | Glutamine and serine rich 1 | Jönsson *et al.* 2011 | |
| RAD9A | RAD9 homologue A (*S. pombe*) | Wright *et al.* 2011 | |
| RGS1 | Regulator of G-protein signalling 1 | Beikler *et al.* 2008 | |
| RGS4 | Regulator of G-protein signalling 4 | Jönsson *et al.* 2011 | |
| RBM25 | RNA binding motif protein 25 | Jönsson *et al.* 2011 | |
| RNASE6 | Ribonuclease, Rnase A family, K6 | Wright *et al.* 2011 | |
| RP1-14N1.3 | Filaggrin 2 | Jönsson *et al.* 2011 | |
| RSAD2 | Radical S-adenosyl methionine domain containing 2 | Jönsson *et al.* 2011 | |
| SAA1 | Serum amyloid A1 | Jönsson *et al.* 2011 | |
| SACS | Spastic ataxia of Charlevoix-Saguenay (sacsin) | Milward *et al.* 2007 | |
| SAMSN1 | SAM domain, SH3 domain and nuclear localization signals 1 | Abe *et al.*2011. | |
| SCARA3 | Scavenger receptor class A member 5 | Abe *et al.*2011. | |
| SCG5 | Secretory granule, neuroendocrine protein 1 (7B2 protein) | Milward *et al.* 2007 | |
| SEC31L1 | SEC31-like 1 (S. cerevisiae) | Milward *et al.* 2007 | |
| SEPHS2 | Selenophosphate synthetase 2 | Wright *et al.* 2011 | |
| SERPINB2 | Serine (or cysteine)proteinase inhibitor, clade B (ovalbumin), member 2 | Milward *et al.* 2007 | |
| Serpine1 | Serpine peptidase inhibitor, clade E (nexin, plasminogen activator inhibitor type 1), member 1 | Beikler *et al.* 2008 | |
| SILV | Silver homolog (mouse) | Jönsson *et al.* 2011 | |
| SLC19A2 | Solute carrier family 19 (thiamine transporter), member 2 | Wright *et al.* 2011 | |
| SOCS1 | Suppressor of cytokine signaling 1 | Beikler *et al.* 2008 | |
| SOD2 | Superoxide dismutase 2, mitochondrial | Milward *et al.* 2007, Wright *et al.* 2011 | |
| SOX9 | SRY (sex determining region Y)-box 9 | Milward *et al.* 2007 | |
| SUI1 | Putatuive translation initiation factor | Wright *et al.* 2011 | |
| TAK1 | TGF-beta activated kinase 1 | Bodet *et al*. 2007. | |
| TANK | TRAF family member-associated NFKB activator | Beikler *et al.* 2008 | |
| TBC1D2 | TBC1 domain family, member 2 | Wright *et al.* 2011 | |
| TBK1 | TANK-binding kinase 1 | Bodet *et al*. 2007. | |
| TIMP1 | TIMP metallopeptidase inhibitor 1 | Bodet *et al*. 2007. | |
| TIMP2 | TIMP metallopeptidase inhibitor 2 | Bodet *et al*. 2007. | |
| TIPARP | TCDD-inducible poly(ADP-ribose) polymerase | Wright *et al.* 2011 | |
| TLR3 | Toll-like receptor 3 | Beikler *et al.* 2008 | |
| TLR2 | Toll-like receptor 2 | Wang *et al.* 2003, Beikler *et al.* 2008 | |
| TLR4 | Toll-like receptor 4 | Wang *et al.* 2003 | |
| TMPRSS2 | Transmembrane protease, serine 2 | Jönsson *et al.* 2011 | |
| TNC | Tenascin C (hexabrachion) | Milward *et al.* 2007 | |
| TNFAIP3 | Tumor necrosis factor, alpha-induced protein 3 | Milward *et al.* 2007, Beikler *et al.* 2008 | |
| TNFA | Tumour necrosis factor-alpha | Wang *et al.* 2003 | |
| TNFRSF9 | Tumor necrosis factor receptor superfamily, member 9 | Beikler *et al.* 2008 | |
| TNFSF10 | Tumor necrosis factor (ligand) superfamily, member 10 | Beikler *et al.* 2008 | |
| TRAF1 | TNF reception-associated factor 1 | Beikler *et al.* 2008 | |
| UBD | Ubiquitin D | Jönsson *et al.* 2011 | |
| UQCRC2 | Ubiquinol-cytochrome c reductase core protein II | Jönsson *et al.* 2011 | |
| UQCRFS1 | Ubiquinol-cytochrome c reductase, Rieske iron-sulphur plypeptide 1 | Wright *et al.* 2011 | |
| USP1 | Ubiquitin specific- peptidase 1 | Jönsson *et al.* 2011 | |
| VCAM1 | Vascular cell adhesion molecule 1 | Beikler *et al.* 2008 | |
| VCAN | Versican | Beikler *et al.* 2008 | |
| VEGF | Vascular endothelial growth factor | Wright *et al.* 2011 | |
| VGF | VGF nerve growth factor inducible | Wright *et al.* 2011 | |
| WIPI1 | WD40 repeat protein Interacting with phospholnositides of 49 kDa | Milward *et al.* 2007 | |
| ZFP91 | Zinc finger protein 91 homolog (mouse) | Jönsson *et al.* 2011 | |

**Table. 3.** List of down-regulated genes in periodontitis with corresponding references.

| Symbol | Gene Name | Source (Paper) |
| --- | --- | --- |
| APCS | Amyloid P component, serum | Beikler *et al.* 2008 |
| ATP10B | ATPase, class V, type 10B | Abe *et al.*2011. |
| BCL2A1 | BCL2-related protein A1 | Beikler *et al.* 2008 |
| BIRC2 | Baculoviral IAP repeat containing 2 | Beikler *et al.* 2008 |
| BIRC3 | Baculoviral IAP repeat containing 3 | Beikler *et al.* 2008 |
| C21orf91 | C21orf91 | Wright *et al.* 2011 |
| C3 | Complement component 3 | Beikler *et al.* 2008 |
| CCL2 | Chemokine (C-C motif) ligand 2 | Beikler *et al.* 2008 |
| CCL3 | Chemokine (C-C motif) ligand 3 | Beikler *et al.* 2008 |
| CCL4 | Chemokine (C-C motif) ligand 4 | Beikler *et al.* 2008 |
| CCL5 | Chemokine (C-C motif) ligand 5 | Beikler *et al.* 2008 |
| CCR1 | Chemokine (C-C motif) receptor 1 | Beikler *et al.* 2008 |
| CCR2A | Chemokine (C-C motif) receptor 2 isoform a | Beikler *et al.* 2008 |
| CCR2B | Chemokine (C-C motif) receptor 2 isoform b | Beikler *et al.* 2008 |
| ITGAX | Integrin, alpha X (complement component 3 receptor 4 subunit) | Beikler *et al.* 2008 |
| CD14 | CD14 molecule | Beikler *et al.* 2008 |
| CD20 | Membrane-spanning 4-domains, subfamily A, member 1 | Beikler *et al.* 2008 |
| CD36 | CD36 molecule | Abe *et al.*2011. |
| CD40 | CD40 molecule, TNF receptor superfamily member 5 | Beikler *et al.* 2008 |
| CD7 | CD7 molecule | Beikler *et al.* 2008 |
| CD8 | CD8 molecule | Beikler *et al.* 2008 |
| CFB | Complement factor B | Beikler *et al.* 2008 |
| CLCA2 | Chloride channel, calcium activated, family member 2 | Milward *et al.* 2007 |
| F3 | - | Beikler *et al.* 2008 |
| COL7A1 | Collagen, type VII, alpha 1 | Beikler *et al.* 2008 |
| COX2 | Cyclooxygenase-2 | Beikler *et al.* 2008 |
| CRIP2 | Cysteine-rich protein 2 | Milward *et al.* 2007 |
| CRP | C-reactive protein, pentraxin-related | Beikler *et al.* 2008 |
| CTGF | Connective tissue growth factor | Beikler *et al.* 2008 |
| CXCL10 | Chemokine (C-X-C motif) ligand 10 | Beikler *et al.* 2008 |
| CXCL5 | Chemokine (C-X-C motif) ligand 5 | Beikler *et al.* 2008 |
| DMKN | Dermokine | Abe *et al.*2011. |
| DSC1 | Desmocollin-1 | Kim *et al.* 2006 |
| EDN1 | Endothelin 1 | Beikler *et al.* 2008 |
| EGFR | Epidermal growth factor receptor | Milward *et al.* 2007, Beikler et al. 2008 |
| EGFR | Epidermal growth factor receptor | Milward *et al.* 2007 |
| EPB42 | Erythroctye membrane protein band4.1 like 4B | Abe *et al.*2011. |
| EPPK1 | Epiplakin | Abe *et al.*2011, Milward *et al.* 2007 |
| FABP4 | Fatty acid binding protein 4, adipocyte | Milward *et al.* 2007 |
| FAT2 | FAT tumor suppressor homolog 2 | Milward *et al.* 2007 |
| FGF2 | Fibroblast growth factor 2 (basic) | Beikler *et al.* 2008 |
| FGFR3 | Fibroblast growth factor receptor 3 | Milward *et al.* 2007 |
| FLJ31821 | Hypothetical protein FLJ31821 | Wright *et al.* 2011 |
| FN1 | Fibronectin | Beikler *et al.* 2008 |
| GBP2 | Guanylate binding protein 2, interferon-inducible | Beikler *et al.* 2008 |
| GCH1 | GTP cyclohydrolase 1 | Beikler *et al.* 2008 |
| GLUL | Glutamate-ammonia ligase (glutamine synthase) | Milward *et al.* 2007 |
| GLUL | Glutamate-ammonia ligase (glutamine synthase) | Milward *et al.* 2007 |
| GM-CSF | colony stimulating factor 2 receptor, beta, low*-*affinity (granulocyte*-*macrophage) | Beikler *et al.* 2008 |
| GPNMB | Glycoprotein (transmembrane) | Milward *et al.* 2007 |
| GTF21 | General transcription factor II, I | Milward *et al.* 2007 |
| HEG1 | HEG homolog 1 (zebrafish) | Milward *et al.* 2007 |
| HIG2 | Hypoxia-inducible protein 2 | Wright *et al.* 2011 |
| HLA-DRB3 | Major histocompatibility complex, classII, DR β3 | Wright *et al.* 2011 |
| HPGD | Hydroxyprostaglandin dehydrogenase 15-(NAD) | Milward *et al.* 2007 |
| HPGD | Hydroxyprostaglandin dehydrogenase 15-(NAD) | Milward *et al.* 2007 |
| HPR | Haptoglobin | Beikler *et al.* 2008 |
| HSXIAPAF1 | XIAP associated factor 1 | Beikler *et al.* 2008 |
| ID3 | Inhibitor of DNA binding 3 | Milward *et al.* 2007 |
| IFIT1 | Interferon-Induced Protein With Tetratricopeptide Repeats | Beikler *et al.* 2008 |
| IFNG | interferon, gamma | Beikler *et al.* 2008 |
| IGFBP2 | Insulin-like growth factor binding protein 2, 36 kDa | Milward *et al.* 2007 |
| IL1RA | Interleukin 1 receptor, type A | Beikler *et al.* 2008 |
| IL15 | Interleukin 15 | Beikler *et al.* 2008 |
| IL1A | Interleukin 1, alpha | Beikler *et al.* 2008 |
| IL1B | Interleukin 1, beta | Beikler *et al.* 2008 |
| IL1F5 | Interleukin 36 receptor antagonist | Beikler *et al.* 2008 |
| IL1F9 | Interleukin 36, gamma | Beikler *et al.* 2008 |
| IL1R | Interleukin 36, gamma | Beikler *et al.* 2008 |
| IL2 | Interleukin 2 | Beikler *et al.* 2008 |
| IL23A | Interleukin 23, α subunit p19 | Wright *et al.* 2011 |
| IL6 | Interleukin 6 (interferon, beta 2) | Beikler *et al.* 2008 |
| IL7R | Interleukin 7 receptor | Beikler *et al.* 2008 |
| IL8 | Interleukin 8 | Beikler *et al.* 2008 |
| IL9 | Interleukin 9 | Beikler *et al.* 2008 |
| IRF1 | Interferon regulatory factor 1 | Beikler *et al.* 2008 |
| IRF7 | Interferon regulatory factor 7 | Beikler *et al.* 2008 |
| ITGB4 | Integrin, beta 4 | Milward *et al.* 2007 |
| ITGB4 | Integrin, beta 4 | Milward *et al.* 2007 |
| JAG2 | Jagged 2 | Milward *et al.* 2007 |
| JAG2 | Jagged 2 | Milward *et al.* 2007 |
| Junb | Transcription factor Junb | Beikler *et al.* 2008 |
| KIAA1068 | K1AA1068 protein | Wright *et al.* 2011 |
| KLK5 | Kallikrein 5 | Milward *et al.* 2007 |
| KRT10 | Keratin 10 | Abe *et al.*2011. |
| KRT13 | Keratin 13 | Milward *et al.* 2007 |
| KRT14 | Keratin 14 | Milward *et al.* 2007 |
| KRT15 | Keratin 15 | Milward *et al.* 2007 |
| KRT17 | Keratin 17 | Abe *et al.*2011. |
| KRT2A | Keratin 2A | Kim *et al.* 2006 |
| KRT4 | Keratin 4 | Milward *et al.* 2007 |
| LOX | Lysyl oxidase | Milward *et al*. 2007 |
| LOX | Lysyl oxidase | Milward *et al.* 2007 |
| LOX | Lysyl oxidase | Milward *et al.* 2007 |
| MARCKS | Myristoylated alanine-rich protein kinase C substrate | Milward *et al.* 2007 |
| MDA5 | Melanoma Differentiation-Associated protein 5 | Beikler *et al.* 2008 |
| METTL7A | DKFZP586A0522 protein | Milward *et al.* 2007 |
| MMP12 | Matrix metalloproteinase 12 (macrophage elastase) | Milward *et al.* 2007, Beikler et al. 2008 |
| MMP13 | Matrix metalloproteinase 13 (collagenase 3) | Milward et al. 2007, Beikler et al. 2008 |
| MMP14 | Matrix metallopeptidase 14 (membrane-inserted) | Beikler *et al.* 2008 |
| MMP15 | Matrix metallopeptidase 15 (membrane-inserted) | Beikler *et al.* 2008 |
| MMP3 | Matrix metallopeptidase 3 (stromelysin 1, progelatinase | Beikler *et al.* 2008 |
| MMP8 | Matrix metallopeptidase 8 (neutrophil collagenase) | Beikler *et al.* 2008 |
| MPPED2 | Chromosome 11 open reading frame 8 | Milward *et al.* 2007 |
| MXRA5 | Matrix-remodelling associated 5 | Milward *et al.* 2007 |
| NEDD9 | Neural precursor cell expressed, developmentally down-regulated 9 | Wright *et al.* 2011 |
| NEFL | Neurofilament, light polypeptide 68 kDa | Milward *et al.* 2007 |
| NFKB1 | Nuclear factor of kappa light polypeptide gene enhancer in B-cells 1 | Beikler *et al.* 2008 |
| NFKBIA | Nuclear factor of kappa light polypeptide gene enhancer in B-cells inhibitor, alpha | Beikler *et al.* 2008 |
| NOS2A | Nitric oxide synthase 2, inducible | Beikler *et al.* 2008 |
| OLFML2A | Olfactomedin-like 2A | Milward *et al.* 2007 |
| PAI2 | Plasminogen activator inhibitor - 1 | Beikler *et al.* 2009 |
| PI3 | Peptidase inhibitor 3, skin-derived | Abe *et al.*2011. |
| PIWIL2 | Piwi-like 2 (Drosophila) | Wright *et al.* 2011 |
| PKP3 | Plakophilin 3 | Milward *et al.* 2007 |
| PLA2 | Phospholipase A2 | Beikler *et al.* 2008 |
| PLAU | Urokinase | Beikler *et al.* 2008 |
| PLAUR | Urokinase Receptor | Beikler *et al.* 2008 |
| POF1B | Premature ovarian failurem 1B | Abe *et al.*2011. |
| PTBP1 | Polypyrimidine tract binding protein 1 | Milward *et al.* 2007 |
| PTGES | Prostaglandin E synthase | Beikler *et al.* 2008 |
| REL | v-rel reticuloendotheliosis viral oncogene homolog (avian) | Beikler *et al.* 2008 |
| RGS2 | Regulator of G-protein signaling 2 | Beikler *et al.* 2008 |
| RIP2 | Receptor-interacting serine-threonine kinase 2 | Beikler *et al.* 2008 |
| RIPK4 | Receptor-interacting serine-threonine kinase 4 | Milward *et al.* 2007 |
| RORA | RAR-related orphan recptor A | Abe *et al.*2011. |
| RPL37A | Ribosomal protein L37a | Milward *et al.* 2007 |
| SAA1 | serum amyloid A1 | Beikler *et al.* 2008 |
| SCD | Stearoyl-CoA desaturase (delta-9-desaturase) | Milward *et al.* 2007 |
| SELE | Selectin E | Beikler *et al.* 2008 |
| SOD2 | Superoxide dismutase 2 | Beikler *et al.* 2008 |
| SREBF1 | Sterol regulatory element binding transcription factor 1 | Wright *et al.* 2011 |
| STON1 | Stoned B-like factor | Milward *et al.* 2007 |
| TG | Thyroglobulin | Wright *et al.* 2011 |
| THBS1 | Thrombospondin 1 | Milward *et al.* 2007 |
| TLR4 | Toll-like receptor 4 | Beikler *et al.* 2008 |
| TM7SF3 | TM7SF3 | Kim *et al.* 2006 |
| TNFAIP1 | Tumor necrosis factor α-induced protein 2 | Wright *et al.* 2011 |
| TNFA | Tumour necrosis factor α | Beikler *et al.* 2008 |
| TNFR | Tumour necrosis factor receptor | Beikler *et al.* 2008 |
| TNFSF13B | Tumor necrosis factor (ligand) superfamily, member 13b | Beikler *et al.* 2008 |
| TRAF1 | TNF receptor-associated factor 1 | Wright *et al.* 2011 |
| TREM1 | Triggering receptor expressed on myeloid cells 1 | Beikler *et al.* 2008 |
| TTLL4 | Tubulin tyrosine ligase-like family, member 4 | Wright *et al.* 2011 |
| VILL | Villin-like | Wright *et al.* 2011 |
| WFDC5 | WAP four-disulfide core domain 5 | Abe *et al.*2011. |

**References**

Abe D, Kubota T, Morozumi T, Shimizu T, Nakasone N, Itagaki M, Yoshie H. 2011. [Altered gene expression in leukocyte transendothelial migration and cell communication pathways in periodontitis-affected gingival tissues.](http://www.ncbi.nlm.nih.gov.ezproxy.liv.ac.uk/pubmed/21382035) *J Periodontal Res* **46,** 345-353.

Beikler T, Peters U, Prior K, Eisenacher M, Flemmig TF. 2008. [Gene expression in periodontal tissues following treatment.](http://www.ncbi.nlm.nih.gov.ezproxy.liv.ac.uk/pubmed/18606014) *BMC Med Genomics* **1**,30.

Bodet C, Andrian E, Tanabe S, Grenier D. 2007. [*Actinobacillus actinomycetemcomitans* lipopolysaccharide regulates matrix metalloproteinase, tissue inhibitors of matrix metalloproteinase, and plasminogen activator production by human gingival fibroblasts: a potential role in connective tissue destruction.](http://www.ncbi.nlm.nih.gov.ezproxy.liv.ac.uk/pubmed/17299802) *J Cell Physiol* **212,** 189-194.

Jönsson D, Ramberg P, Demmer RT, Kebschull M, Dahlén G, Papapanou PN. 2011. [Gingival tissue transcriptomes in experimental gingivitis.](http://www.ncbi.nlm.nih.gov.ezproxy.liv.ac.uk/pubmed/21501207) *J Clinl Periodontol* **38,** 599-611.

Kim DM, Ramoni MF, Nevins M, Fiorellini JP. 2006. [The gene expression profile in refractory periodontitis patients.](http://www.ncbi.nlm.nih.gov.ezproxy.liv.ac.uk/pubmed/16734580) *J Periodontol* **77**, 1043-1050.

Milward MR, Chapple IL, Wright HJ, Millard JL, Matthews JB, Cooper PR. 2007. [Differential activation of NF-kappaB and gene expression in oral epithelial cells by periodontal pathogens.](http://www.ncbi.nlm.nih.gov.ezproxy.liv.ac.uk/pubmed/17355248) *Clin Exp Immunol* **148,** 307-324.

Tanabe S, Bodet C, Grenier D. 2008. [Treponema denticola lipooligosaccharide activates gingival fibroblasts and upregulates inflammatory mediator production.](http://www.ncbi.nlm.nih.gov.ezproxy.liv.ac.uk/pubmed/18366071) *J Cell Physiol* **216,** 727-731.

Wang PL, Ohura K, Fujii T, Oido-Mori M, Kowashi Y, Kikuchi M, Suetsugu Y, Tanaka J. 2003. [DNA microarray analysis of human gingival fibroblasts from healthy and inflammatory gingival tissues.](http://www.ncbi.nlm.nih.gov.ezproxy.liv.ac.uk/pubmed/12767925) *Biochem Biophys Res Commun* **305,** 970-973.

Wright HJ, Chapple IL, Matthews JB, Cooper PR. 2011. [Fusobacterium nucleatum regulation of neutrophil transcription.](http://www.ncbi.nlm.nih.gov.ezproxy.liv.ac.uk/pubmed/20663022) *J Periodontal Res* **46**, 1-12.
